# Melanosome transfer to keratinocyte in the chicken embryonic skin is mediated by vesicle release associated with Rho-regulated membrane blebbing

**DOI:** 10.1038/srep38277

**Published:** 2016-12-02

**Authors:** Ryosuke Tadokoro, Hidetaka Murai, Ken-ichiro Sakai, Takahiro Okui, Yasuhiro Yokota, Yoshiko Takahashi

**Affiliations:** 1 Department of Zoology, Graduate School of Science, Kyoto University, Kitashirakawa, Sakyo-ku, Kyoto, 606-8502, Japan; 2 Graduate School of Biological Sciences, Nara Institute of Science and Technology, Takayama, Ikoma, Nara, 630-0192, Japan; 3 AMED Core Research for Evolutional Science and Technology (AMED-CREST), Japan Agency for Medical Research and Development (AMED), Chiyoda-ku, Tokyo 100-0004, Japan

## Abstract

During skin pigmentation in amniotes, melanin synthesized in the melanocyte is transferred to keratinocytes by a particle called the melanosome. Previous studies, mostly using dissociated cultured cells, have proposed several different models that explain how the melanosome transfer is achieved. Here, using a technique that labels the plasma membrane of melanocytes within a three-dimensional system that mimics natural tissues, we have visualized the plasma membrane of melanocytes with EGFP in chicken embryonic skin. Confocal time-lapse microscopy reveals that the melanosome transfer is mediated, at least in part, by vesicles produced by plasma membrane. Unexpectedly, the vesicle release is accompanied by the membrane blebbing of melanocytes. Blebs that have encapsulated a melanosome are pinched off to become vesicles, and these melanosome-containing vesicles are finally engulfed by neighboring keratinocytes. For both the membrane blebbing and vesicle release, Rho small GTPase is essential. We further show that the membrane vesicle-mediated melanosome transfer plays a significant role in the skin pigmentation. Given that the skin pigmentation in inter-feather spaces in chickens is similar to that in inter-hair spaces of humans, our findings should have important consequences in cosmetic medicine.

Skin pigmentation is essential to protect the body against ultraviolet irradiation^1^. In amniotes (birds and mammals), melanin, a main component of skin pigmentation, is synthesized in a specialized organelle called melanosome in the melanocyte. The melanosome is transported to the tip of a melanocyte dendrite, a long cellular process, via the cytoskeleton^2^,^3^,^4^,^5^, and is subsequently translocated to adjacent keratinocytes, which do not produce melanin. Thus, the melanocyte-to-keratinocyte transfer of melanosomes (thereafter called melanosome transfer) is an essential process that covers a wide surface of the skin with melanin pigmentation, and therefore the mechanisms of melanosome-transfer have been a central issue in dermatology, cosmetology, and cell biology.

The melanosome transfer has been conventionally studied either by electron microscopy and/or *in vitro* analyses using co-culture with dissociated keratinocytes and melanocytes. Studies using these techniques have proposed three prominent models to explain how the melanosome transfer is achieved^6^,^7^,^8^,^9^,^10^,^11^,^12^,^13^,^14^,^15^,^16^,^17^,^18^,^19^,^20^,^21^. First model is that the melanosome-containing tip of a dendrite or filopodium protruding from melanocytes is directly cytophagocytosed by adjacent keratinocytes. These observations were made using guinea pig cells^8^ and human cells^10^, and the model is corroborated by the finding that the inhibition of myosin X, which is required for the formation of filopodia, disturbs the melanosome transfer^15^.

Second model highlights that a melanosome is released from melanocytes as a plasma membrane-enveloped vesicle, which is subsequently incorporated into keratinocytes by phagocytosis. Such phenomena were shown using *Xenopus* cells^12^, human melanoma cells^9^, and human foreskin cells^16^,^17^. This model was corroborated by Wu *et al*.^18^, who showed in time-lapse analyses of *in vitro* culture cells taken from the skin of transgenic mice that keratinocytes retained melanosome-containing membrane vesicles that had been transferred from the melanocytes. The proposal that keratinocytes received their melanin through membrane vesicle-mediated transfer was also supported by a recent study using atomic force microscopy^20^.

The third model proposes that melanin pigments are exocytosed to an extracellular space by fusion of melanosome membrane and plasma membrane. This model has been supported by electron microscopies showing naked melanin pigments in intercellular space^6^,^11^. Tarafder *et al*. also suggested that exocytosis of melanosomes occurring in cultured human skin cells is regulated by Rab11b^21^. Other models have also been casted including a tunneling nanotube of plasma membrane through which melanosomes are transmitted^22^.

Basically, these postulated mechanisms are each based on the behaviors of cells proliferating in 2-D culture; and it is increasingly appreciated that cellular behaviors in a 2-D environment could profoundly be different from those cultured in 3-D or *in vivo*^23^,^24^,^25^. For instance, whereas *in vitro* cultured tumor cells are tightly attached to the culture dish with actin stress fibers firmly extended, these cells undergo different behaviors when placed in 3-D environment such as Matrigel, where cytoskeletons are flexibly rearranged with associated plasma membrane blebbing^23^,^24^,^25^. However, while it is important to know how melanosome transfer is achieved in the actual body, there are, at present, no experimental systems that allow direct visualization of melanocytes’ behavior, particularly in amniotes.

In this study, we established time-lapse live imaging of developing melanocytes using chicken embryos. At early stages, neural crest cells, the melanocyte precursors, are electroporated with GFP genes. From these embryos at later stages, a skin tissue retaining the 3-D integrity was dissected and subjected to the live-imaging analyses. We observed that GFP-melanocytes released membrane vesicles, each of which contained a single (not multiple) melanosome, which were ultimately incorporated into keratinocytes. This partially confirms previous *in vitro* studies. To our surprise, however, the release of membrane vesicles was accompanied by the dynamic blebbing of the plasma membrane, which has previously been unappreciated. Furthermore, we found that both the membrane blebbing and membrane vesicle production were regulated by Rho protein activity. We show that this mode of melanosome transfer by melanocyte membrane blebbing and vesicle production is essential for skin pigmentation.

## Results

**Figure 1:**
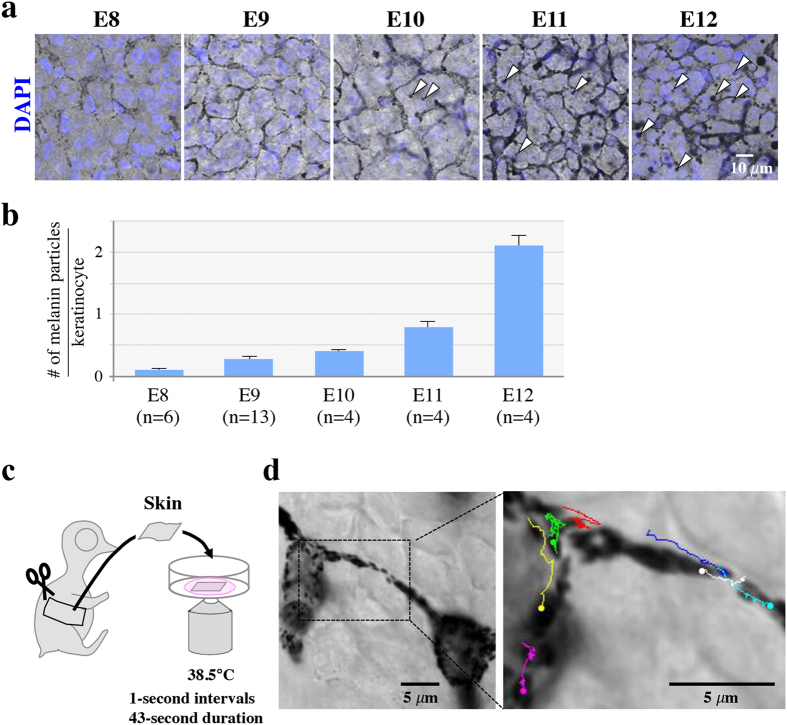
Melanosome transfer to neighboring keratinocytes in the skin of chicken (Hypeco nera) embryos. (**a**) Melanocyte maturation and pigmentation during skin development. Melanocytes are represented as black dendritic cells colored by melanin pigments. Keratinocytes surrounding melanocytes are shown as white cells stained nucleus with DAPI. Arrowheads indicate melanin particles in keratinocytes. (**b**) Average number of transferred-melanin particles per keratinocyte. 400 keratinocytes were quantified in each embryo. Data are presented as the mean ± SEM. (**c**) *Ex vivo* live imaging using a skin of E8 chicken embryo. A skin tissue dissected from embryos was placed in a glass bottom dish with culture medium containing agarose gel, and was immediately observed by confocal microscope. (**d**) Time-lapse imaging (bright field) revealed melanosomes actively moving in melanocytes. Colored lines indicate trajectories of melanosomes, which were tracked manually using Image J (1 second-intervals, 43 second-duration) (Supplementary video 1).

We started our analyses to determine when the melanosome transfer starts during chicken development. We peeled a piece of embryonic skin containing both the epidermis and dermis from a flank region of embryos at different developmental stages, and prepared flat-mounted specimens of the skin (Fig. 1a). At embryonic day 8 (E8), melanocytes were recognized as pigmented cells, but no melanosomes were found in neighboring keratinocytes (Fig. 1a,b). By E11, in contrast, melanosomes started to be detected in keratinocytes, and the number of the melanosomes increased onward (Fig. 1a,b). Thus, the melanosome transfer must take place around E11–E12. A skin piece of E8 embryos was further subjected to time-lapse microscopy after placing it in a glass-bottom dish embedded in agarose-containing medium (Fig. 1c). Bright field time-lapse observation for 43 seconds showed melanosomes moving actively within a melanocyte (Fig. 1d, Supplementary video 1).

**Figure 2:**
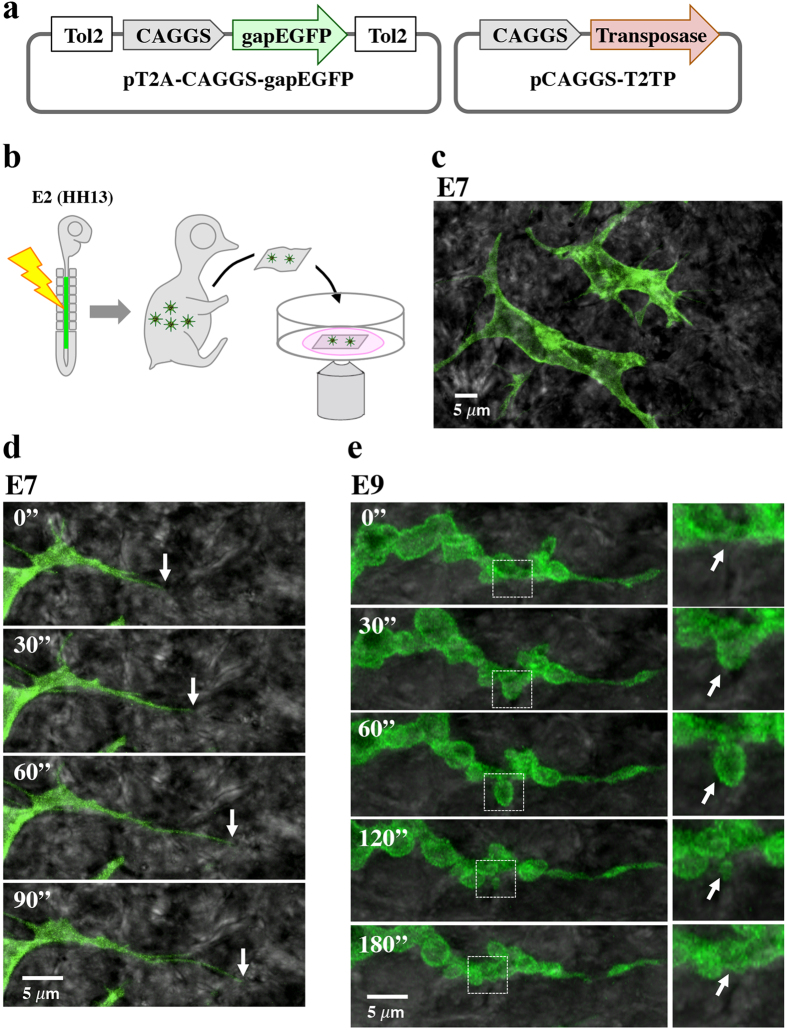
Gene manipulation and high-resolution imaging analyses of melanocyte precursors. (**a**) Plasmids used for stable transgenesis of neural crest/melanocytes by Tol2-mediated gene transfer. (**b**) Experimental procedure for confocal microscopy with gapEGFP-labeled melanocytes in the skin of Hypeco nera embryos. Tol2-gapEGFP gene was electroporated into the neural tube/neural crest of E2 (HH13) embryos. From manipulated embryos of E7, E9 or E12, a skin was pealed off and placed in a glass bottom dish with agarose-containing culture medium, and was observed by confocal microscope. (**c**) gapEGFP-labeled melanocytes in the skin at E7. (**d** and **e**) Representative images selected from movies (Supplementary video 2,3,4) showing dynamic changes in dendrite behaviors at E7 (**d**) and E9 (**e**). Images were obtained using the confocal microscope Carl Zeiss LSM5 PASCAL. White arrows indicate the tip of dendrite (E7) and bleb (E9), respectively.

For high-resolution live imaging analyses, EGFP-labeling of the plasma membrane of melanocytes was necessary. To overcome the low efficiency of gene transfection into differentiated melanocytes, we used the in ovo DNA electroporation into neural crest cells, the precursors of melanocytes^26^,^27^,^28^. In addition, the Tol2 transposon-mediated gene transfer technique was used enabling stable expression of electroporated EGFP cDNA^29^,^30^. Thus, two kinds of plasmids were electroporated into neural crest cells at E2: one was pT2A-CAGGS-gapEGFP (pT2A: Tol2 transposon-carrying vector; gapEGFP: membrane tethered form of EGFP), and the other was pCAGGS-T2TP (T2TP: transposase) (Fig. 2a). Electroporated embryos were subjected to the aforementioned skin preparation, followed by confocal time-lapse microscopy at E7 (HH 30), E9 (HH 35), or E12 (HH 38) (Fig. 2b).

At E7, gapEGFP-labeled melanocyte precursors exhibited active protrusion of dendrites and filopodia that repeatedly extended and retracted. Some of the dendrites extended 20 μm long (Fig. 2c,d, Supplementary video 2 and 3). At E9, the behavior of melanocyte precursors was drastically changed: the dendrites harbored multiple bubble-like structures emerging from the plasma membrane, the phenomenon known as membrane blebbing (Fig. 2e, Supplementary video 4)^31^.

**Figure 3:**
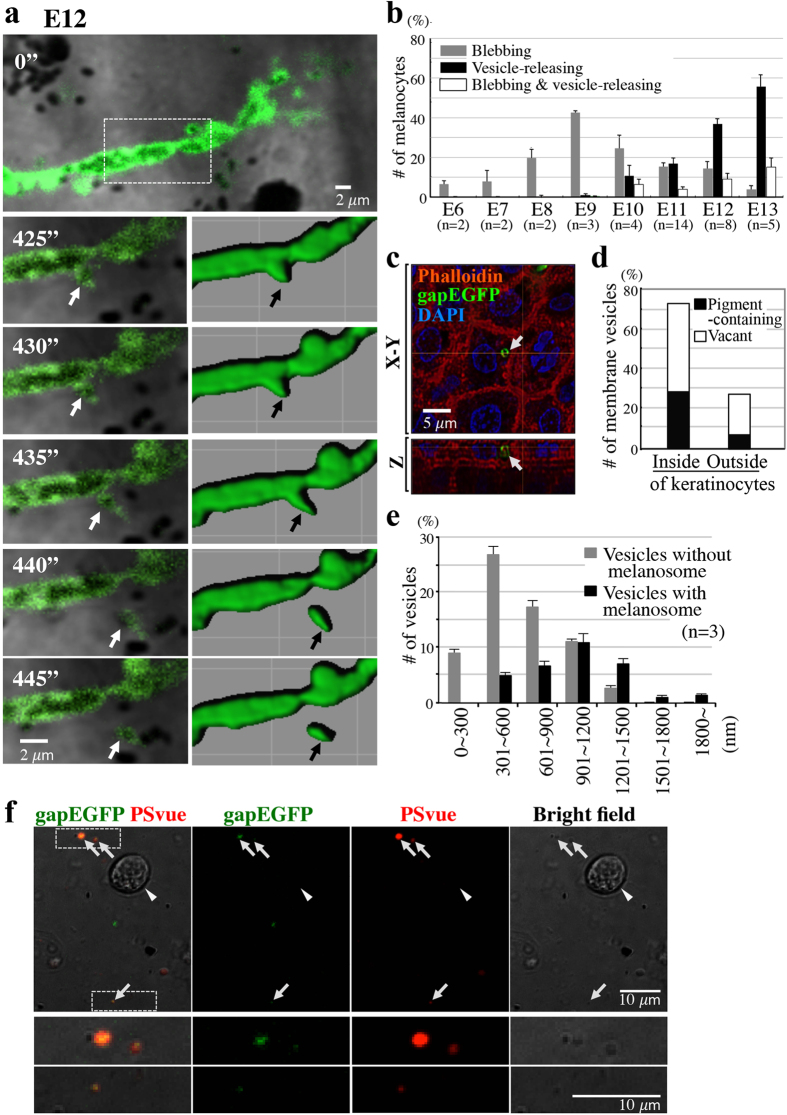
Melanosome transfer is mediated by plasma membrane vesicles. (**a**) A melanocyte dendrite at E12. Lower panels show magnified images of a square selected from time-lapse movies by Carl Zeiss LSM5 PASCAL (Supplementary video 5), and surface-rendering images by IMARIS 7.6 (Bitplane). Arrows show a membrane bleb that was eventually released from the dendrite. (**b**) From bleb to vesicle changes of the melanocyte plasma membrane during development. Blebbing cells emerge around E6 onward, followed by appearance of vesicle-releasing melanocytes around E10. 30 melanocytes were assessed for each embryo. The definition was made by morphological criteria of membrane tethered EGFP-expressing melanocytes using high-resolution images obtained by confocal microscopy. We define the vesicle-releasing melanocytes as the cells adjacent to which released EGFP-positive vesicles are observed. Blebbing melanocytes are those that exhibit blebs in the plasma membrane. And the rest of the cells are classified as non-blebbing melanocytes. Data are presented as the mean ± SEM. (**c**) An orthogonal confocal image (Nikon A1R) shows an EGFP-positive membrane vesicle (arrow) incorporated into a keratinocyte, whose shape was visualized by phalloidin staining. (**d**) Among 185 vesicles examined in E12 embryos, a majority was found within keratinocytes. In each fraction of inside and outside keratinocytes, the number of pigment-containing vesicles was smaller than that of vacant vesicles. (**e**) A range of different sizes of membrane vesicles with and without a melanosome. 30 vesicles were assessed for each of three embryos (E12). Data are presented as the mean ± SEM. (**f**) Melanocyte-derived gapEGFP+ membrane vesicles are enriched with phosphatidylserine in the outer lipid bilayer (arrows). Membrane vesicles were isolated from the skin of E12 embryos, and subjected to staining with PSvue 550 (red) (See Material and Methods). Lower panels show magnified images of boxed areas in the upper panels.

By E12, some of the membrane blebs were pinched off from the melanocyte, and released as small vesicles, followed by a translocation into keratinocytes. Figure 3a and Supplementary video 5 show sequential steps of such vesicle release. Importantly, some of the discharged vesicles contained a melanosome (Fig. 3a,e, Supplementary video 5). The onset of the vesicle release (E10–E11) was consistent with the developmental stage when melanosomes started to be detected in keratinocytes (Figs 1a and 3b).

To determine if all the released vesicles were translocated into the keratinocytes, the keratinocytes were visualized by either phalloidin staining or transfection with the gap-mOrange gene (Fig. 3c, Supplementary Figure S1)^32^. By confocal microscopy with Z-stack analyses, 72.8% of discharged vesicles were found within the keratinocytes whereas 27.2% of vesicles remained in extracellular spaces (Fig. 3c,d). In each fraction, the number of melanosome-containing vesicles was smaller than that of vacant vesicles: 39.7% and 60.3% inside keratinocytes, and 25.5% and 74.5% outside keratinocytes, respectively (Fig. 3d), implying that the melanosome is not requisite for the vesicles to enter the keratinocytes. The vesicles located within the keratinocyte were often enclosed by a phagosome-like structure visualized by gap-mOrange-labeled plasma membrane of keratinocytes (Supplementary Figure S1). Thus, melanosome-containing vesicles discharged from the melanocytes are engulfed by keratinocytes through phagocytosis.

A majority of the discharged membrane vesicles were less than 1,500 nm in diameter (Fig. 3e), and therefore classified as plasma membrane vesicles (also called shedding vesicles, microparticles) distinct from exosomes that are much smaller in size^33^,^34^. Since not all the membrane vesicles contained a melanosome (Fig. 3e), it is unlikely that melanosome’s encapsulation by a vesicle is a trigger of the vesicle release. Although the blebbing has been reported to be characteristic of apoptotic cells^35^,^36^, we detected little sign of apoptosis during melanocyte differentiation (Supplementary Figure S2). We also found that the outer layer of released vesicles was enriched with phosphatidylserine revealed by PSvue staining *in vitro* with freshly prepared vesicles from E12 embryos (Fig. 3f).

**Figure 4:**
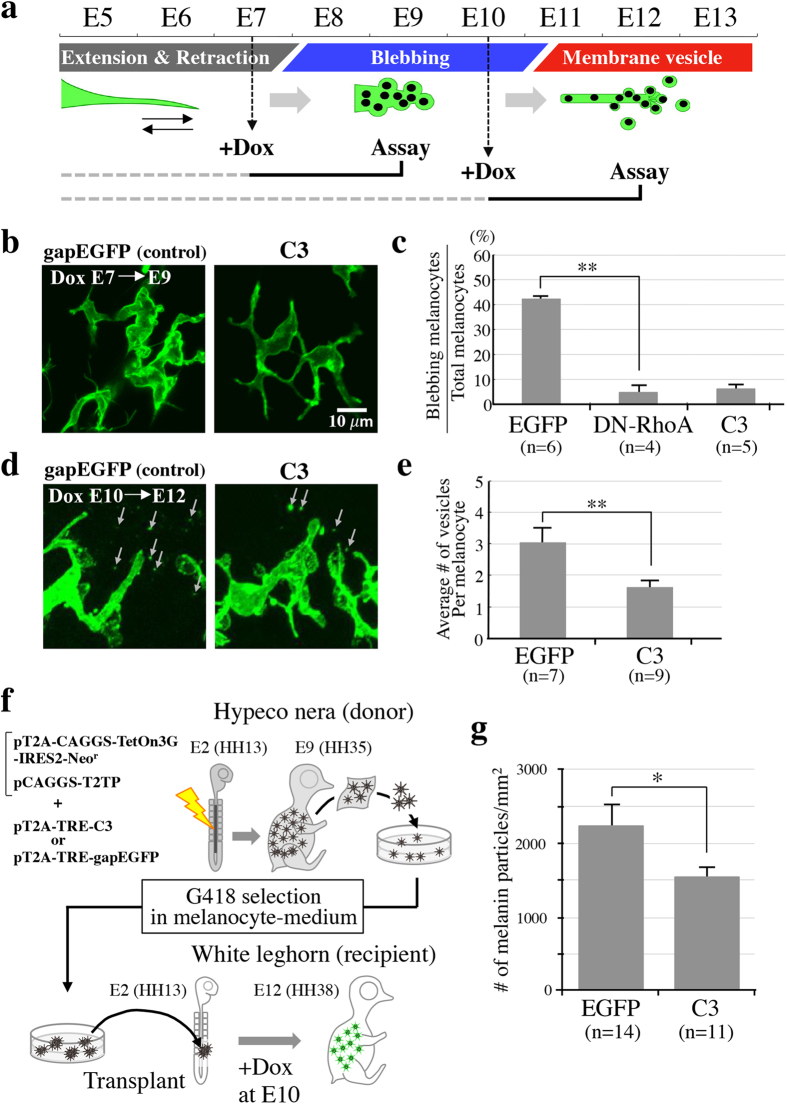
Rho activity is required for the membrane vesicle formation and skin pigmentation. (**a**) Temporally controlled inhibition of Rho activity using the tet-on system. Doxycycline (Dox) was administered into embryos at E7 and E10 to turn on the DN-RhoA- or C3 genes prior to the stages when blebbing and vesicle release would normally start. Effects by Rho inhibition for respective events were evaluated at the stages indicated. (**b**–**e**) Rho inhibition resulted in a marked reduction of both membrane blebbing (**b**,**c**) and release of membrane vesicles (arrows) (**d**,**e**). The photos in (**b**) are selected images from Supplementary video 6 (control gapEGFP) and Video 7 (C3). (**f**) Experimental procedure for pigmentation assay. pT2A-CAGGS-TetOn3G-IRES-Neo^r^, pCAGGS-T2TP, and pT2A-TRE-gapEGFP/pT2A-TRE-GAPEGFP-C3 genes were electroporated into the neural tube of Hypeco nera (pigmented strain) at E2. A skin tissue taken from E9 embryo was dissociated into single cells, and gene-electroporated melanocytes were enriched in G418-containing culture medium. Subsequently, an aggregate of enriched melanocytes was transplanted into host embryos of White leghorn (non-pigmented) at E2. The C3 gene turned on by Dox administration at E10. See Material and Methods. (**g**) Melanin particles in 1 mm^2^ of the skin were quantified by Nikon software NIS-elements. All values of statistical data are shown as the mean ± SEM. Statistical significance was calculated using Student’s t-test: *P < 0.05, **P < 0.005.

We further explored the molecular mechanisms by which the membrane blebbing and the vesicles release are regulated. It has previously been reported that platelets and invasive cancer cells undergo membrane blebbing and vesicle release, dependent on the activities of members of the small GTPase Rho family^31^,^34^,^37^. To determine whether Rho signaling would also be important for the membrane vesicle-mediated transfer of melanosomes, Rho-inhibiting constructs, either dominant negative RhoA (DN-RhoA) or botulinum C3, were electroporated into the developing neural crest cells/melanocyte lineage. Expression of these genes was temporally controlled using the tet-on inducible system^38^. To evaluate the effects of Rho-inhibition on the membrane blebbing and vesicle release, doxycycline (analog of tetracycline) was administered to embryos at E7 and E10, respectively (Fig. 4a).

When RhoA activity was inhibited at E7, the membrane blebbing was markedly reduced by E9 (Fig. 4b, Supplementary video 6 and 7). Dendrites of E9 melanocytes introduced with DN-RhoA or C3 were smoother than those of control with a reduced amount of membrane blebs. Statistical analyses also confirmed this observation: whereas the relative number of control EGFP-electroporated cells with membrane blebbing over total melanocytes was 42.5 +/− 0.8%, this was reduced to 4.5 +/− 2.7% (in DN-RhoA-treated cells) and 5.9 +/− 1.9% (in C3-treated cells) (Fig. 4c). The importance of Rho signals for the blebbing was corroborated by a pharmaceutical treatment with Y27632 (Rock inhibitor) that resulted in not only a reduction of the blebs but also a generation of filopodia that would normally be seen in immature melanocytes (Fig. 2d, Supplementary video 8).

We also found that RhoA was important for the release of membrane vesicles from melanocytes. Activation of DN-RhoA or C3 at E10 resulted in a significant reduction of vesicle release assessed at E12 (Fig. 4d,e). Whereas the average number of released vesicles around an EGFP-control melanocyte was 3.0 +/− 0.5, this was markedly reduced to 1.6 +/− 0.2 when C3 was expressed (Fig. 4e). However, during the investigation we noticed that the extent of vesicle release implementation was highly variable even among normal embryos. To more precisely scrutinize the effect of RhoA inhibition on the vesicle release, we assessed the effects in a single embryo. To do so, we electroporated cDNAs of mStrawberry (control) and C3/RockDN in separated melanocyte-forming regions of a single embryo. In E2 embryos, a drop of mineral oil was laid in the lumen of neural tube, followed by in ovo electroporation with mStrawberry in the anterior region to the oil and C3 or RockDN in the posterior region (Supplementary Figure S3a). Since the oil drop prevented mixing of DNA solutions in the neural tube, the treated embryo had mStrawberry-positive melanocytes in the anterior skin and C3- or RockDN-positive melanocytes in the posterior with a clear boundary between them (Supplementary Figure S3a). Similar to the experiment shown in Fig. 4e, C3 was activated by Dox at E10, and the effects were assessed at E12. In control embryos where melanocytes in the anterior and posterior halves were electroporated with mStrawberry (ST) and EGFP, respectively, a ratio of the number of released membrane vesicles was 1.20 +/− 0.19 (EGFP/ST) (Supplementary Figure S3c). By contrast, when Rho-A activity was inhibited in the posterior skin, the ratio was markedly reduced to 0.57 +/− 0.05 (C3/ST), and 0.43 +/− 0.07 (RockDN/ST) (Supplementary Figure S3b,c). Together, we conclude that the RhoA-Rock signaling is critical for both the membrane blebbing and vesicle release during melanocyte maturation.

Finally, we examined whether the inhibition of vesicle release would affect the skin pigmentation. Since the efficiency of DNA electroporation used in above experiments was not high enough to cause a detectable change in skin pigmentation, we used the recently established technique of melanocyte transplantation, which enables gene manipulation in cells of high density^32^. Briefly, G418 DNA-electroporated neural crest cells of the Hypeco nera (pigmented) strain of chicken are placed in culture, followed by enrichment of G418-resistant melanocyte precursors in melanocyte growth medium (Fig. 4f; see Material and Methods)^32^. Subsequently, these cells are transplanted into a host embryo of White leghorn (unpigmented), where the Hypeco nera-derived cells populate and behave as normal melanocytes^32^. In this study, we electroporated pT2A-TRE-C3 (tet-inducible) and pT2A-CAGGS-TetOn3G-IRES-Neo^r^ (tet-non-inducible; TetOn3G encodes tet-dependent transcriptional activator) along with pCAGGS-T2TP (Tol2 transposase) into Hypeco nera embryos (Fig. 4f). Following the enrichment of G418^r^-melanocyte precursors in culture and a transplantation of these cells into a host embryo of White leghorn, Dox was administered at E10, and the embryos were assessed for pigmentation at E12 (Fig. 4f). The number of melanin particles per 1 mm^2^ of control EGFP-expressing skin area was 2,211 +/− 290 (n = 14), whereas that of C3 was 1,545 +/− 129 (Fig. 4G; n = 11). Together, we conclude that the membrane vesicle release from the melanocytes contributes profoundly to the skin pigmentation, and also that the melanosome transfer to keratinocytes is mediated, at least in part, by membrane vesicle protrusion and its release in the chicken embryo.

## Discussion

We have demonstrated that the melanosome transfer in the chicken 3-D skin is mediated, at least in part, by plasma membrane vesicles. Melanosome-containing vesicles are discharged from a melanocyte into extracellular spaces, and subsequently engulfed by neighboring keratinocytes. Our findings are consistent with one of the three prevailing models proposed by *in vitro* studies^6^,^7^,^8^,^9^,^10^,^11^,^12^,^13^,^14^,^15^,^16^,^17^,^18^,^19^,^20^,^21^ (See also Introduction). Of course, our study does not exclude the other models of cytophagocytosis- and exocytosis-mediated transfer^6^,^7^,^8^,^10^,^11^,^15^,^21^, and it is indeed underway to see if melanocytes in different locations of the body send their melanosomes in a different way. We have also demonstrated that differentiating melanocytes undergo blebbing of the plasma membrane prior to the vesicle release. Some of the melanosome-encapsulated blebs are pinched off to become vesicles. We have finally shown that RhoA plays important roles in both the membrane blebbing and vesicle release. Thus, the membrane vesicle-mediated transfer of melanosome is a consequence of multiple steps during melanocyte differentiation. The delineation of these steps has been enabled by high amenability of chicken embryos, in which melanocyte precursors/neural crest cells can specifically be gene-manipulated in 3-D environment.

What triggers the melanosome encapsulation in the membrane vesicles? Since more than half of discharged vesicles are “vacant” (Fig. 3e), it is likely that the melanosome encapsulation is a stochastic event. Whereas no vesicles contain multiple melanosomes, numerous melanosomes are recognized in a single keratinocyte, implying that these melanosomes might derive from different vesicles and from different melanocytes. Indeed, we have observed GFP-labeled and –unlabeled vesicles in a single keratinocyte (Supplementary video 5). Likewise, it is conceivable that melanosomes produced by a single melanocyte are delivered to multiple keratinocytes, thus enabling a wide coverage of the skin with melanin particles. Importantly, the membrane vesicle-mediated transfer plays a significant role in the skin pigmentation since inhibition of the vesicle production results in a reduced amount of membrane blebbing and pigmentation in the body skin (Fig. 4f,g). The membrane vesicle-mediated transfer of melanosomes that we have described in this study appears to be distinct from that reported by Wu *et al*., who showed that relatively large vesicles are pinched off in which multiple melanosomes are packed^18^. It is possible that different mechanisms exist in the body for the production of membrane vesicles.

High-resolution live imaging analyses combined with membrane-tethered EGFP labeling of melanocyte precursors have revealed that the membrane blebbing precedes the vesicle production. Considering that the vesicles are the product of these blebs, the blebbing must be requisite for the membrane vesicle-mediated melanosome transfer. Membrane blebbing has not been reported by previous studies using *in vitro* cultured melanocytes, raising the possibility that the membrane dynamics of melanocytes might be regulated by 3-D environment. We have also observed that the plasma membrane of discharged vesicles exhibit a phosphatidylserine-enriched outer layer, the well known “eat me” signal recognized by neighboring cells shown in phagocytosis of apoptotic cells (Fig. 3f)^39^. It is likely that this flipped localization of phosphatidylserine already occurs in the blebbing plasma membrane.

Since the membrane blebbing and vesicle release have been reported for platelets and several invasive cancer cells^40^,^41^,^42^, one can speculate that the membrane vesicle-mediated intercellular communications might take place in broader types of cells than previously thought. Relevant to this, melanoma cells have been reported to produce protease-containing membrane vesicles that might be used to degrade extracellular matrices for their metastasis^43^. It is conceivable that the membrane vesicles produced by melanocytes (this study) might also serve as a vehicle to convey not only a melanosome but also other substances. Exosomes (particles smaller in size than the membrane vesicle) produced by some cancer cells contain signal substances such as microRNAs, and are delivered to a long-distance target^44^.

Another important finding obtained in this study is that RhoA activity is critical for both the membrane blebbing and vesicle release in maturing melanocytes revealed by temporally controlled knockdown experiments (Fig. 4). It is widely accepted that Rho and Rac activities counteract each other in a variety of intracellular events^45^,^46^,^47^. We speculate that RhoA activity might be localized to the blebbing site whereas the rest of plasma membrane maintains its integrity by Rac activity. This notion is consistent with the previous study that Rac1 is important for the dendrite formation in melanocytes^48^, and also with our finding that the inhibition of RhoA in E9 melanocytes resulted not only in the reduction of blebbing but also in promoting filopodia (Supplementary Figure S4, and Supplementary Video 8).

The regulation of skin pigmentation is critical not only for protection against solar radiation, but also for camouflage and individual identification leading to survival and reproductive strategies in a wide variety of animals. In humans, particularly, the pigmentation is an important cosmetic concern in numerous syndromes involving depigmentation. It should be noted that the melanocytes in chickens are seen both in between and around feather buds, and this pattern of distribution is similar to humans, but not to mice where melanocytes are restricted to hair follicles. The molecular and cellular mechanisms unveiled in this study will open the way to further investigations into how the skin pigmentation emerged during animal evolution, and may also ultimately have important consequences for medicine.

## Materials and Methods

### Ethical approval

All animal experiments were performed in accordance with the methods and protocols approved by the institutional animal care and use committees of Kyoto University (No. H2716, Kyoto University).

### Chicken strains

Fertilized eggs of Hypeco nera and White leghorn were purchased from Shiroyama poultry farm (Kanagawa, Japan) and Takeuchi poultry farm (Nara, Japan), respectively. Embryos were staged according to Hamburger Hamilton^49^. Embryonic day 2 (E2) corresponds to HH13, and E6, E7, E8, E9, E10, E11, E12, and E13 to HH29, HH30, HH34, HH35, HH36, HH37, HH38, and HH39, respectively.

### Phalloidin and nucleus staining of chicken embryonic skin

Embryos were fixed in 4% (w/v) paraformaldehyde (PFA) at 4 °C for 12 hours, and a piece of the skin was peeled from the flank region of embryos. Specimens were treated with PBST (phosphate-buffered saline with 0.1% (v/v) Tween 20) at 4 °C. To detect filamentous actin and nucleus, specimens were subjected to a reaction with Alexa Flour 568 phalloidin (Thermo Fisher), and 4′, 6-Diamidino-2-phenylindole (DAPI) (DAPI solution 1 mg/ml, Dojindo) (phalloidin 1/300 and DAPI 1/1000 dilution in PBST) for 14 hours at room temperature. After washing in PBST at room temperature, specimens were sealed by Fluor Save reagent. Microscopic images were obtained using the confocal laser-scanning microscopy Nikon A1R.

### Time-lapse imaging of melanocytes in a skin of chicken embryo

A piece of skin tissue containing the epidermis, dermis, and subcutaneous components was stripped off from the muscle layer of chicken embryos in a pre-warmed Dulbecco’s Modified Eagle Medium (DMEM) at 38.5 °C using micro scissors (Napox MB54-2). The stripped piece was placed in a glass-bottom dish (CELLview^TM^ 627860, Greiner bio one) with the epidermal side facing the bottom. The specimen was covered by a polyethylene terephthalate membrane (Cell Culture Insert, BD falcon 3090) to avoid distortion of the tissue, and embedded in 1% low-melting agarose gel (Ultrapure LMP Agarose, Invitrogen) containing the medium (DMEM, HAM/F12, 2 mM L-Glutamine, 2 mM Sodium Pyruvate, Penicillin-Streptomycin). After the gel setting, culture medium (DMEM/HamF12 supplemented with L-Glutamine, phenol red, 0.26 mM Glucose, 0.25 mM Sodium bicarbonate, 0.15 mM HEPES, and B-27 supplement) was carefully added on the gel surface. The specimens were observed by the confocal microscope Nikon A1R or Carl Zeiss LSM510 at 38.5 °C.

### Constructions of vectors

The open reading frame (ORF) of GAP43-EGFP (gapEGFP) provided by Dr. Nakagawa (Hokkaido Univ.), was subcloned into the EcoRI-XhoI site of pT2A-CAGGS^32^. pT2A-CAGGS-Tet3G: The ORF of Tet3G isolated from pCMV-Tet3G (Clontech) was subcloned into the EcoRI-BglII site of pT2A-CAGGS. pCAGGS-T2TP was previously described^30^. pT2A-BI-TRE-gapEGFP: The pT2AL200R150G vector provided by Dr. Kawakami (NIG) was digested with BglII-XhoI to remove the cassette containing EF1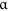 promoter-EGFP-SV40polyA^29^. This site was blunt-ended, and inserted with the fragment of pT2K-BI-TRE-gapEGFP, which contains bidirectional tetracycline-responsive element (TRE) with two minimal promoters of CMV in both directions, and the polyA-additional sequence of the rabbit beta globin gene. pT2A-BI-TRE-gapmStrawberry: The ORF of mStrawberry was PCR-amplified from pmStrawberry (Clontech). The product of mStrawberry was inserted into the SalI-BspEI site of pT2A-BI-TRE-gapEGFP, from which EGFP was removed. pT2A-BI-TRE-gapEGFP- (C3, DN-RhoA or DN-Rock): The ORFs of either C3 transferase, a dominant negative form of RhoA (DN-RhoA), or a dominant negative form of Rock2 (DN-Rock), provided by Dr. Kaibuchi (Nagoya Univ.), was subcloned into the MluI-EcoRV site of pT2A-BI-TRE-gapEGFP. pT2A-CAGGS-Tet3G-IRES2-Neo^r^: The oligonucleotide encoding IRES2 and the ORF of neomycin resistant gene (Neo^r^) were PCR-amplified from pIRES2-EGFP and pIRES-Neo^r^ (Clontech), respectively. The products of IRES2 and Neo^r^ were inserted into the EcoRI-EcoRV site of pT2A-CAGGS. This pT2A-CAGGS vector carrying IRES2 and Neo^r^ was digested with EcoRI, followed by blunt-ended. This site was inserted with the ORF of Tet3G. RCASBP-gap-mOrange: The oligonucleotide encoding a membrane localization signal of GAP43 (gap) was purchased from Operon Biotechnologies. The ORF of mOrange was PCR-amplified from pmOrange vector (Clontech). The products of GAP and mOrange were cloned into PstI-HindIII site of pSLAX13 shuttle vector, followed by subcloning of a ClaI-product into the RCASBP-A retrovirus vector.

### In ovo electroporation

The in ovo electroporation was performed as previously described^26^,^27^,^28^ with slight modifications. A DNA solution was prepared at 4 μg/μl (Stable expression; pT2A-CAGGS-gapEGFP: pCAGGS-T2TP = 3: 1, Conditional expression; pT2A-BI-TRE-gapEGFP: pT2A-CAGGS-Tet3G: pCAGGS-T2TP = 2: 1: 1), and injected into the lumen of neural tube of E2 (HH13) embryos of Hypeco nera. An electric pulse of 30 V, 1 ms, was given, followed by 5 times of pulses of 8 V, 25 ms, with 475 ms intervals (BEX, Pulse generator CUY21EX).

### Administration of a doxycycline (Dox) solution for temporally regulated manipulations of Rho-Rock activities

As previously described^38^, 500 μl of Dox solution (0.3 mg/ml in HANKS: 140 mM NaCl, 5.4 mM KCl, 5.6 mM glucose, 0.34 mM Na_2_HPO_4_, 10 mM HEPES, 1 mM MgCl_2_, 1 mM CaCl_2_ pH 7.0) was injected into the yolk using 1 ml-syringe (27-G needle).

### Pigmentation assay

Enrichment of gene-introduced melanocytes: A dorsal skin was dissected from E9 embryos using micro-scissors, and treated with 0.25% (v/v) trypsin/ethylenediaminetetraacetic acid (EDTA) for 30 min at 38.5 °C. A suspension of dissociate cells was placed in Primaria^TM^ 60 mm cell culture dishes (BD Biosciences) coated with 50 μg/ml recombinant fibronectin (Wako) according to the optimized culture condition for melanocytes^50^. One day after incubation, melanocytes were fed with Mel-mix medium containing 10 ng/ml 12-o-tetradecanoylphorbol-13-acetate (TPA, Wako) and 500 μg/ml G418 (Wako) to select melanocytes expressing Tet3G and Neo^r^. A fresh Mel-mix medium containing TPA and G418 was added to culture every 3 days. These cells were cultured at 38.5 °C at 5% CO_2_. The procedures were also described previously^32^.

Transplantation of cultured melanocytes into White leghorn embryos: Cell aggregates of melanocytes were prepared with the hanging drop method^51^. Melanocytes-containing medium (8 μl) was dropped on the lid of petri dish (BD Biosciences), and DMEM was placed in the bottom of counterpart dish to keep moist. The drops were incubated for 24–48 hours in an incubator supplied with 5% CO_2_, 38.5 °C, until a cell aggregate formed at the tip of the drop. For the transplantation, an epidermal ectoderm at the 20^th^ to 25^th^ somite level of White leghorn embryos was incised using a sharpened tungsten needle, and a melanocyte aggregate was placed into the incision using a glass needle as previously described in ref. 32. The treated embryos were re-incubated until E10 when a Dox solution was administered, followed by re-incubation until E12. Melanin particles in these specimens were observed by the confocal microscope NikonA1R, and quantified by Nikon software Nis-elements.

### Vesicle isolation and phosphatidylserine staining

Skin tissues containing gapEGFP^+^-melanocytes of E12 embryos were dissociated by treatment with 0.25% (v/v) trypsin/EDTA for 30 min at 38.5 °C. The cell suspension was centrifuged at 20,000 g for 30 min. The precipitation containing cells and vesicles were washed in DMEM/HamF12 medium, and resuspended in 100 μl of the medium. 1 μl of PSvue550 solution (5 mM) was mixed with an equal volume of 10.5 mM zinc nitrate solution and incubated at room temperature for 30 min. This mixture was diluted 2.5-fold with water, and 1 μl of the diluted solution was added to the vesicle containing medium.

## Additional Information

**How to cite this article**: Tadokoro, R. *et al*. Melanosome transfer to keratinocyte in the chicken embryonic skin is mediated by vesicle release associated with Rho-regulated membrane blebbing. *Sci. Rep.* **6**, 38277; doi: 10.1038/srep38277 (2016).

**Publisher's note:** Springer Nature remains neutral with regard to jurisdictional claims in published maps and institutional affiliations.

## Supplementary Material

Supplementary Video 1

Supplementary Video 2

Supplementary Video 3

Supplementary Video 4

Supplementary Video 5

Supplementary Video 6

Supplementary Video 7

Supplementary Video 8

Supplementary Video 9

Supplementary Video 10

Supplementary Information
