# Supplementary material for: Melanosome transfer to keratinocyte in the chicken embryonic skin is mediated by vesicle release associated with Rho-regulated membrane blebbing
Source: Sci Rep. 2016 Dec 2;6:38277. doi: 10.1038/srep38277 (PMC5133614; doi:10.1038/srep38277)
Supplement: Supplementary Information [file srep38277-s11.pdf]

Supplementary information for

Melanosome transfer to keratinocyte in the chicken embryonic skin is mediated by vesicle release associated with Rho-regulated membrane blebbing

Ryosuke Tadokoro[1], Hidetaka Murai[1, 2, 3], Ken-ichiro Sakai[2], Takahiro Okui[2] Yasuhiro Yokota[2, 4] and Yoshiko Takahashi[1, 5]*

[1] Department of Zoology, Graduate School of Science, Kyoto University, Kitashirakawa, Sakyo-ku, Kyoto, 606-8502, Japan

[2] Graduate School of Biological Sciences, Nara Institute of Science and Technology, Takayama, Ikoma, NARA, 630-0192, Japan

[3]Present address: Frontier Research Institute for Interdisciplinary Sciences (FRIS) Tohoku University, Aoba-ku, Sendai, 980-8578, Japan

[4]Present address: National Cardiovascular Center Research Institute, Fujishirodai, Suita, Osaka 565-8565, Japan

[5]AMED Core Research for Evolutional Science and Technology (AMED-CREST), Japan Agency for Medical Research and Development (AMED), Chiyoda-ku, Tokyo 100-0004, Japan

*Author for correspondence:

Yoshiko Takahashi, Professor

Department of Zoology, Graduate School of Science, Kyoto University,

yotayota@develop.zool.kyoto-u.ac.jp

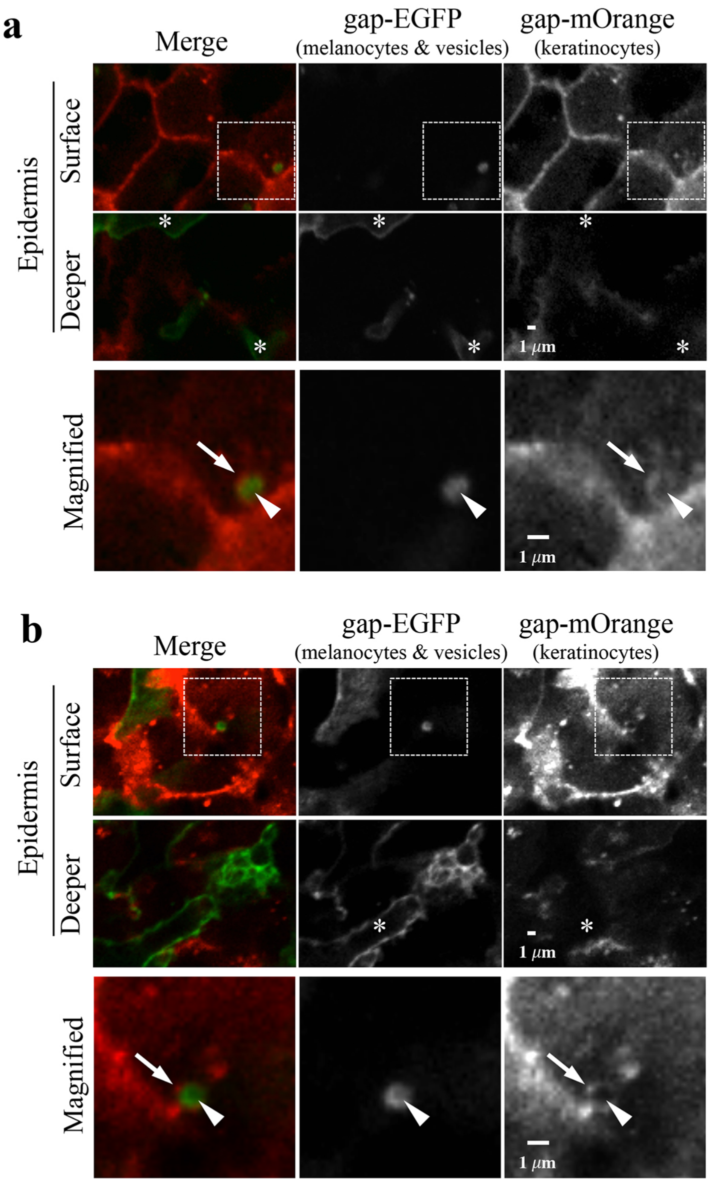

Supplementary Figure S1

Membrane vesicles incorporated into keratinocytes are enclosed by keratinocyte-derived phagosome-like structures (arrows). a and b) The surface and deeper layers of epidermis of E12 chicken embryos. Lower pictures represent magnified images of the boxed areas of the upper pictures. Keratinocytes are labeled with gap-mOrange by using the retrovirus-mediated infection method (See methods) [32]. Gap-EGFP is seen in melanocytes and melanocyte-derived membrane vesicles, which are gap-mOrange-negative (asterisks). EGFP+ membrane vesicles (arrowheads) were enclosed by mOrange-positive phagosome-like structure (arrows). Images were captured by confocal microscope Nikon A1R with GaAsP detectors.

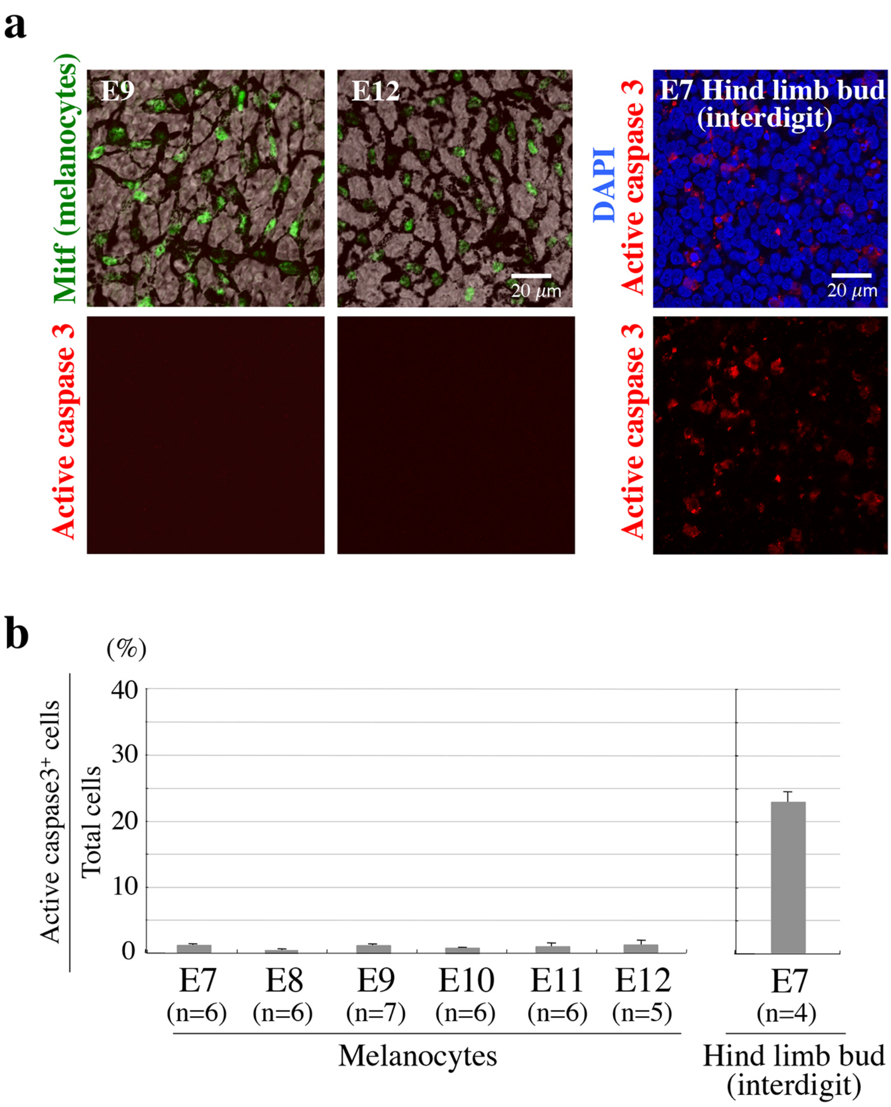

Supplementary Figure S2

No sign of apoptosis during melanocyte maturation in the skin. (a) Immunostaining for active caspase 3 in the skin. Interdigital cells of hind limb bud was used as a positive control. (b) Quantification of active caspase 3-positive cells. Statistical data are shown as the mean ± SEM.

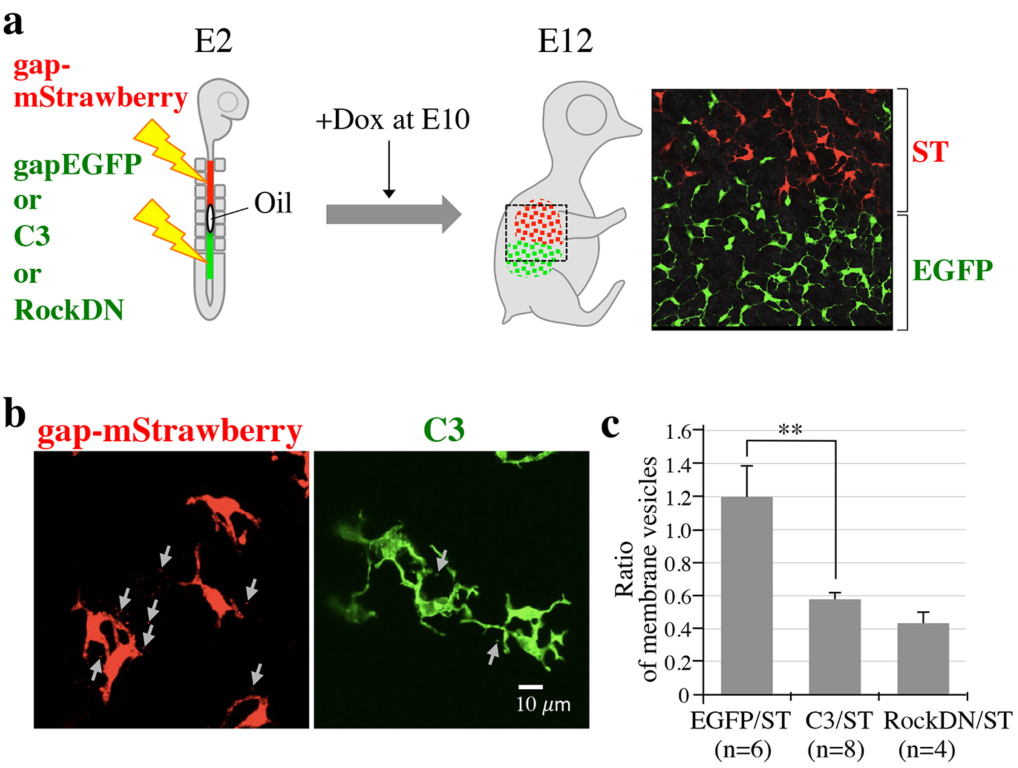

Supplementary Figure S3

Simultaneous manipulations of control- and Rho-inhibitory genes in a single embryo corroborating the findings obtained in Fig. 4. (a) To avoid variability among individuals in membrane vesicle release and pigmentation, control gap-mStrawberry DNA and Rho-inhibitory gene (C3 or Rock DN) were electroporated in the anterior and posterior regions of E2 embryos, respectively. To avoid a mixing of DNA solutions in the lumen of neural tube, an oil drop was laid in the lumen prior to electroporation. In the treated E12 embryo, melanocytes receiving either gene were separately localized with a sharp boundary. The C3- and Rock DN genes were under tet-inducible promoter, turning on by Dox administration. (b) In such treated embryos, C3-expressing melanocytes (posterior) produced a smaller number of membrane vesicles (arrows) than that of gap-mStrawberry-electroporated cells (anterior). (c) A ratio of the number of membrane vesicles produced by C3- or Rock DN-electroporated melanocytes (posterior) over that by control gap-mStrawberry cells (anterior). Values of statistical data are shown as the mean ± SEM. Statistical significance was calculated using Student's t-test: $**P<0.005$.

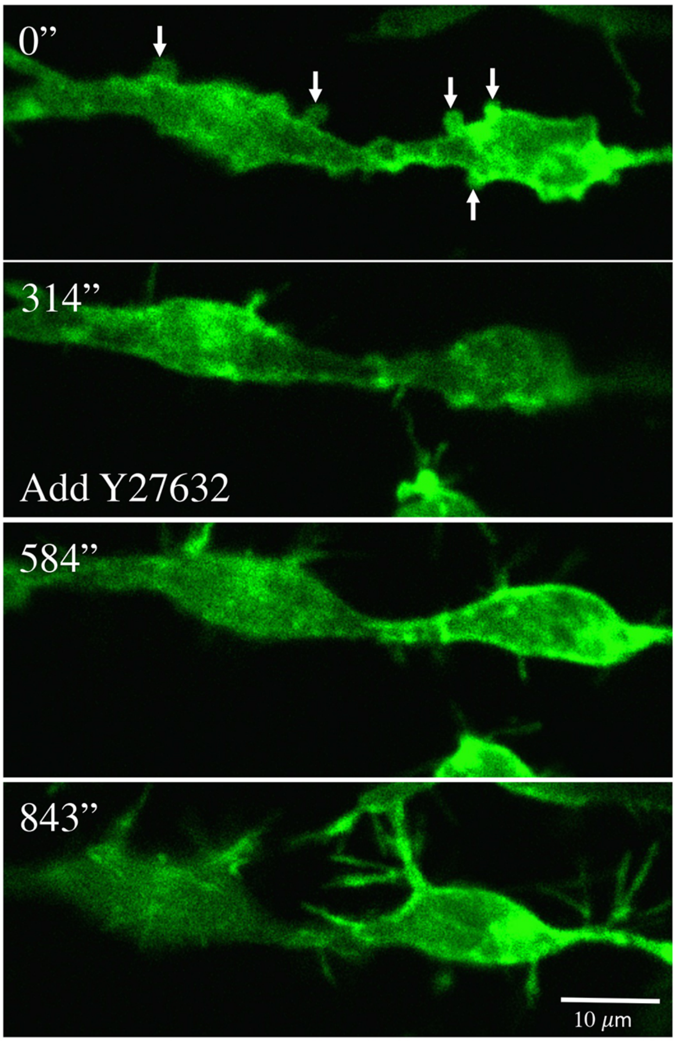

Supplementary Figure S4

Adding Y27632 (time 314’’) to blebbing melanocytes at E9 in culture ceased the blebbing, but instead enhanced filopodia protrusion. Arrows show membrane blebs. Images are selected from Video 8.

Methods

Immunohistochemistry

Immunohistochemistry of a flat-mounted skin was performed as follows. Embryos were fixed in 4% PFA at 4 °C for 12 hours, and a piece of the skin was peeled from the flank region. Specimens were treated with 5 % (v/v) FBS/PBST (phosphate-buffered saline with 0.1 % (v/v) Tween20) at 4 °C for 20 hours, and reacted with anti-active caspase 3 antibody (promega; diluted 1:200 in PBST) or with anti-MITF antibody (Clone C5+D5, Invitrogen; diluted 1 : 200 in PBST) over night at 4 °C. After washing in PBST at 4 °C overnight, specimens were reacted with Alexa Fluor 555-conjugated anti-rabbit IgG secondary antibody (Invitrogen; diluted 1:500 in PBST) at room temperature for 1 hour. Microscopic images were obtained by the confocal laser-scanning microscope Nikon A1R.

The "Oil electroporation" method to introduce multiple genes separately into different regions of a single embryo

A small drop of mineral oil (Sigma M8410) was laid into a lumen of neural tube at the 20th somite level of E2 (HH13) embryos by a glass capillary. Subsequently, two sets of DNA solutions, [pT2A-BI-TRE-gapmStrawberry : pT2A-CAGGS-Tet3G : pCAGGS-T2TP = 2 : 1 : 1] and [pT2A-BI-TRE-gapEGFP- (C3 or DN-Rock) : pT2A-CAGGS-Tet3G : pCAGGS-T2TP = 2 : 1 : 1] were separately injected into the neural tube either anteriorly or posteriorly to the oil drop, respectively, followed by electroporation as described in Materials and Methods.

Retrovirus-mediated infection of gap-mOrange gene into keratinocytes

RCASBP-gap-mOrange retrovirus vector was transfected into the chicken cell line DF1 using Lipofectamine 2000 (Invitrogen). DF1 cells were cultivated in 10 cm cell culture dishes in DMEM supplemented with 10% (v/v) fetal bovine serum (FBS) to confluence at 38.5°C, 5% CO_2. Supernatants containing virus particles were pooled, and filtered through a 0.45 μm polyvinylidene fluoride filter (PALL life sciences). RCAS retroviral particles were concentrated by polyethylene glycol (PEG) precipitation (8% (w/v) Polyethylene glycol 6000, 100 mmol/L NaCl, and 10 mmol/L HEPES, pH 7.5). The PEG-containing suspension was centrifuged at 1500 g at 4°C for 30 min. The precipitate was washed three times and suspended in Opti-MEM (Invitrogen). Viral titer

was determined as described previously ($>1x10^6$ infectious units/mL) [1]. 2.5 μ l of virus working solution (virus solution : polybrene (10 mg/mL) = 10 : 1) was injected into an embryo of White leghorn at E2. The embryo was subsequently transplanted with EGFP-expressing melanocytes taken from Hypeco nera. Details of the procedures were described in “pigmentation assay” and our previous report [32].

Administration of Rock inhibitor

10 μl rock inhibitor Y-27632 (Final concentration: 10 μM diluted in the culture medium for time-lapse) was gently added to the skin tissue embedded in the agarose gel during the time-lapse imaging.

Captions for videos:

Video 1: Movement of melanosomes within melanocytes at E8 embryo. Frames were obtained for 43 seconds with 1-second intervals. Seven colored lines show trajectories of melanosomes, which were manually tracked using image J (NIH, Washington, DC, USA). This video corresponds to Figure 1d.

Video 2: Actively motile melanocytes in a skin of E7 embryo visualized by gapEGFP. Each frame in time-lapse is of a maximum intensity projection of 7 focal planes acquired with 0.5 mm Z-intervals. Frames were captured with 15-second intervals for 600 seconds. This video corresponds to Figure 2c.

Video 3: Enlarged view of the tip of dendrites shown in Video 2 acquired in the same time-scale. Dendrites undergo marked elongations and retractions. This video corresponds to Figure 2d.

Video 4: Plasma membrane blebbing seen in a dendrite of gapEGFP-labeled melanocytes in a skin of E9 embryo. Frames were captured with 30-second intervals for 630 seconds. This video corresponds to Figure 2e.

Video 5: Membrane vesicles containing a melanosome are discharged from a dendrite of E12 melanocyte. Red arrow indicates a membrane bleb that is eventually released as a vesicle. Note that this vesicle contains a melanosome. Time-lapse images were acquired with 5-second intervals for 850 seconds. This video corresponds to Figure 3a.

Video 6: E9 melanocytes with EGFP-expression used as a control experiment for Rho inhibition (Video 7). Time-lapse images were captured with 10-second intervals for 190 seconds. This video corresponds to Figure 4b.

Video 7: E9 melanocytes induced to express C3 at E7. Membrane blebbing was markedly reduced. Time-lapse images were captured with 10-second intervals for 190 seconds. This video corresponds to Figure 4b.

Video 8: Adding Rock inhibitor Y27632 (at time 314’’) to E9 blebbing melanocytes extinguished the blebbing, but instead enhanced filopodia protrusion. Time-lapse images were acquired with 20 second-intervals for 820 seconds. This video corresponds to Supplementary Figure S4.

References:

1. Smith, C. A. et al. The avian Z-linked gene DMRT1 is required for male sex determination in the chicken. Nature 461, 267ï 271, doi: 10.1038/nature08298 (2009).
